# Interferon Genes Are Influenced by 17β-Estradiol in SLE

**DOI:** 10.3389/fimmu.2021.725325

**Published:** 2021-10-18

**Authors:** Ram P. Singh, Bevra H. Hahn, David S. Bischoff

**Affiliations:** ^1^ Research Service, Veteran Administration Greater Los Angeles Healthcare System, Los Angeles, CA, United States; ^2^ Division of Rheumatology, Department of Medicine, University of California, Los Angeles, Los Angeles, CA, United States; ^3^ Department of Medicine, University of California, Los Angeles, Los Angeles, CA, United States

**Keywords:** estradiol, interferon genes, systemic lupus erythematosus, cytokines/chemokines, T cells

## Abstract

Recent evidence suggests the existence of a nexus between inflammatory pathways and the female sex hormone 17β-estradiol, resulting in increased interferon-stimulated genes (ISGs), autoantibodies, and dysregulation of immune cells in SLE. However, the molecular mechanisms and the effect of estradiol on candidate target genes and their pathways remains poorly understood. Our previous work suggests that female SLE patients have increased estradiol levels compared to healthy controls. In the present study, we explored the effects of 17β-estradiol treatment on expression of IFN (interferons)-stimulated genes and pro-inflammatory cytokines/chemokines. We found significantly increased (5-10-fold) expression of IFN-regulated genes in healthy females. Furthermore, we found significantly increased plasma levels of IL-6, IL-12, IL-17, IL-18, stem cell factor (SCF), and IL-21/IL-23 in SLE patients compared to healthy controls, and those levels positively correlated with the plasma levels of 17β-estradiol. In addition, levels of IL-21 positively correlated with the SLE disease activity index (SLEDAI) score of SLE patients. *In vitro* treatment of PBMCs from either SLE patients or healthy controls with 17β-estradiol at physiological concentration (~50 pg/ml) also significantly increased secretion of many pro-inflammatory cytokines and chemokines (IL-6, IL-12, IL-17, IL-8, IFN-γ; MIP1α, and MIP1β) in both groups. Further our data revealed that 17β-estradiol significantly increased the percentage of CD3^+^CD69^+^ and CD3^+^IFNγ^+^ T cells; whereas, simultaneous addition of 17β-estradiol and an ERα inhibitor prevented this effect. Collectively, our findings indicate that 17β-estradiol participates in the induction of pro-inflammatory cytokines and chemokines and further influences interferon genes and pathways.

## Introduction

Systemic lupus erythematosus (SLE) is a chronic autoimmune disease associated with pathogenic autoantibodies and increased levels of type 1 interferon (IFNs) and their signaling molecules both in mice and in humans (1–10). Recently 17β-estradiol has been shown to amplify the activation of IFNα signaling in B cells (11). In addition, previous studies suggest that exacerbation of SLE is more common during pre-menstrual periods and during pregnancy, in which women experience increased estrogen levels (12). Others have suggested that SLE is mediated by autoantibodies and estrogen has been shown to stimulate antibody production by B cells (13). Altogether these studies suggest the existence of a nexus between inflammatory pathways and 17β-estradiol, resulting in increased IFN-stimulated genes (ISGs) and autoantibodies in SLE patients.

In women, treatment with 17β-estradiol-containing medications increases risk for SLE and clinical flares (14–17). Estrogen promotes SLE in part by expanding autoreactive marginal zone B cells and by altering helper T cell activation *via* calcineurin pathways (18, 19), thus promoting pro-inflammatory cytokine generation and T/B cell activation (20–23). The role of type 1 IFNs and the female sex hormone 17β-estradiol in the pathogenesis of autoimmunity has been described earlier (4, 5, 10). One study has identified the estrogen receptor alpha (ER-α) gene (ESR1) as a target induced by both IFNα and IFNγ in distinct cell types, including splenic cells, from lupus mice (24). ER-α (NZBxNZW) F1 knockout female mice do not develop glomerulonephritis (25), and estrogen treatments have been shown to exacerbate the disease and increase mortality (26). In contrast, another study showed that ER-α^-/-^ mice (lacking ER-α in the C57BL/6 background) but not those lacking ER-β (ER-β^-/-^ mice) exhibit immune complex-type glomerulonephritis, proteinuria, and destruction of tubular cells with severe infiltration of B lymphocytes in the kidney and the presence of anti-DNA antibodies in the serum (27). Thus, the role of ER-α in SLE in mice is not completely clear. The interaction among type I and type II IFNs and 17β-estradiol in the regulation of immune response genes expressed in the peripheral blood of SLE patients has been reported (28). The study showed effects of IFNα co-stimulation with either TNF, IFNγ, or E2. TNF has repressive effects while IFNγ has synergistic effects on IFN gene expression *in vitro*. Additionally, the cross-regulation of TNFα on IFNα in autoimmune diseases including SLE patients has been shown (29). The exact mechanism by which 17β-estradiol interacts with IFN in SLE is poorly understood. Further these studies did not identify pathways that may be involved in molecular regulation. The identification and characterization of the molecular mechanisms underlying estradiol’s induction of candidate target genes remains to be elucidated in SLE. Recently we reported that female SLE patients have significantly increased plasma estradiol levels compared to healthy controls. We also found that testosterone levels were decreased in female SLE patients compared to healthy female controls (30). In the present study, we tested the concept that increased amount of 17β-estradiol predisposes women to lupus by driving activation of pro-inflammatory pathways that relate to production of type 1 IFN, generation of Th17 cells by IL-21/23, generation of Th1 cells by IL-12, and regulation of IFN pathways and genes.

## Materials and Methods

### Subjects

We enrolled 14 subjects who were 18 years or older and fulfilled the American College of Rheumatology revised criteria for the classification of SLE (31, 32) and 14 healthy donors (19-70 years of age) with no history of autoimmune disease. Subjects’ characteristics including age, sex, clinical parameters, medications and SLEDAI score are shown in **Table 1**. Subjects had regular menstrual cycles (occurring monthly and lasting 2 to 6 days) and were not taking any contraceptives or sex hormones (estrogen, progesterone, androgen, or testosterone). Irregular menstrual cycle was a criterion for exclusion from the study. Only patients with stable disease activity (SLEDAI <6 and not >6 for past 2 visits) using immunosuppressive drugs, such as glucocorticoids and mycophenolate (1-2 g/day) at stable doses for the past two months, and daily prednisone doses not to exceed 10-15 mg/day, were recruited for the study. Patients with comorbid conditions, such as a. patients with renal dysfunction (serum Cr ≥1.8); b. pregnant women; c. uncontrolled non-lupus medical disease that might affect peripheral T cells; d. patients or controls receiving sex hormone therapies, including DHEA, were excluded from the study. Disease activity was recorded based on the SLE disease activity index (SLEDAI) (33). For estradiol and cytokines measurement, we obtained control and SLE plasma samples from the UCLA Rheumatology Biobank. The study was approved by the Institutional Review Board of the University of California Los Angeles. Written informed consent was obtained from each subject who participated in the study.

**Table 1:** SLE patient demographics, clinical parameters, medications and disease characteristics including SLEDAI score.

Age, Mean (SD)	Sex, Female/Male	Ethnicity	ESR	CRP	ANA	Anti-dsDNAab	SLEDAI	Medications
38.5 (15.27)	Female (78.65%) Male (21.45%)	Asian (14.3%) Hispanic (21.4%), White (64.3%)	38.5 (15.3)	0.60 (0.28)	8/14+ ve	368.4 (317.5)	6.57 (3.20)	Patients were on prednisone, hydroxychloroquine, methotrexate, plaquenil, Imuran, folic acid, Vitamin D, topomax Cellcept (mycophenolate mofetil), furosemide

Data are presented as medians, means (SD) or number (%) as indicated. Age range was between 20-72 years, ESR (Erythrocyte sedimentation rate) range was 3-31, CRP (C- reactive protein) range was in between 0.5-1.5, ANA (Anti-nuclear antibody) was positive in 8 patients out of 14. 6 had < 1:40, Anti-dsDNA Ab (Anti-double strand DNA Ab) range was between 200-1243, SLEDAI (SLE disease activity index) range was between1-10. Medications listed are for all the combined patients. Healthy controls had similar demographics including age range and had no medications at the time of blood draw.

### Mice

NZBxNZW F1 (H-2d/z) mice were purchased from the Jackson Laboratories (Bar Harbor, ME, USA) or bred at the University of California Los Angeles (UCLA). Male and female mice were housed under pathogen-free conditions. All mice were treated in accordance with the guidelines of the University of California Los Angeles Animal Research Committee, an Institution accredited by the Association for Assessment and Accreditation of Laboratory Animal Care (AAALAC).

### Spleen Cells Isolation, RNA Isolation and Real-Time PCR

Spleen cells were isolated from 8-10-week-old male and female BWF1 mice and single cell suspensions prepared by passing cells through cell strainers (Fisher). ACK lysing buffer (Sigma, St Louis, MO, USA) was used to lyse and remove red blood cells. White blood cells were lysed with TRIzol (Invitrogen Inc., Carlsbad, CA) and RNA isolated as per the manufacturer’s protocol. Real-time PCR was performed with 100 ng of RNA from each sample with rodent *IFI202b*-specific primers and probe (Applied Biosystems, Foster City, CA, USA). All values were normalized to GAPDH levels.

### Cell Isolation and Preparation

Peripheral blood mononuclear cells (PBMCs) were isolated on a density gradient (Histopaque-1077, Sigma-Aldrich, St. Louis, MO, USA) from blood samples of lupus patients and healthy volunteers. Lymphocytes were washed twice in serum free media. Red blood cells (RBC) were lysed with RBC lysing solution (Sigma-Aldrich, St. Louis, MO, USA).

### Cell Culture, 17β-Estradiol Treatment, and Flow Cytometry Analyses

PBMCs from SLE patients and healthy controls (4x10^6^ cells) were cultured with 17β-estradiol (50 pg/ml; Sigma-Aldrich, St. Louis, MO, USA) for 24-48 hours range in complete media containing fetal calf serum. After culture, supernatants were collected and cells washed and lysed for RNA analyses. For immunophenotyping flow cytometry study, SLE patients and healthy control PBMCs (2x10^6^) were obtained and treated with 17β-estradiol (0, 10, 50 pg/ml) for 24 hours in serum-free media in the presence or absence of the 17β-estradiol receptor α antagonist ICI-182780 (10 pg/ml; TOCRIS, Minneapolis, MN, USA). Cells were washed twice, stained with anti-CD3 (SK7), anti-CD69 (L78), and anti-IFNγ (25723.11) antibodies and analyzed by FACS. 20 µl of antibody was used for the staining of the cells as per manufacturer’s protocol (BD Biosciences, San Jose, CA). Dead cells were excluded from the analyses based on FSC (forward scattering) and SSC (side scattering) and only live cells were used for the analyses. For intracellular IFNγ staining, cells were first fixed, permeabilized (as per manufacturer’s protocol; BD Biosciences, San Jose, CA), and stained. Cells were washed two times with PBS and data were acquired at the UCLA Flow Cytometry Core Facility. FACS Calibur™, BD LSR II, BD FACS Aria II instruments were used for data acquisition. FACS antibodies were from BioLegend, eBiosciences, or BD Biosciences (San Jose, CA, USA). Data were analyzed using FCS Express™ De Novo software (Ontario, Canada).

### Measurement of Estradiol, Cytokines/Chemokines, and Myriad RBM Human MAPs (Multi-Analyte Profile) Assays

Cytokines, chemokines, and other biomarkers were analyzed from the culture supernatant from 17β-estradiol treated or non-treated PBMCs from SLE patients and healthy controls by RBM multiplexed immunoassay analysis (Myriad RBM Inc., Austin, TX, USA) following the manufacturer’s protocol. 100 ul of supernatant was used in these assays. Human IL-6, IL-12, IL-17A, IL-18, IL-21, and IL-23 were measured by ELISA kit from BioLegend (San Diego, CA, USA). Estradiol levels were measured in plasma and culture supernatants by commercial ELISA (Calbiotech Inc., Spring Valley, CA) as per manufacturer’s instructions.

### RNA Isolation, Gene Expression, and Real Time PCR Analyses

RNA was isolated from cultured PBMCs with TRIzol (Invitrogen Inc., Carlsbad, CA). Gene expression analyses of candidate target genes were analyzed and real-time PCR performed as described earlier (34–38). Quantitative one-step real-time PCR was performed using TaqMan technology (100 ng of total RNA) on an ABI Prism 7900 HT Sequence Detection System (Applied Biosystems, Foster City, CA, USA, *OASL, LY6E, IFI202b*, and *GAPDH* primers and probes were obtained from Applied Biosystems.

### Statistical Analyses

Data was analyzed using Prism 4.0 (GraphPad Software, San Diego, CA). Comparisons between two groups were performed using paired one- or two-tailed Student’s t test. One and two- way ANOVA analysis was performed for more than two-grouped data sets. Linear regression analysis was performed to correlate 17β-estradiol levels with IL-6 and IL-21 levels. Results are expressed as mean ± SEM. p<0.05 was considered significant.

## Results

### Healthy Females Have Significantly Higher Interferon Regulated Genes

**Figure 1:**
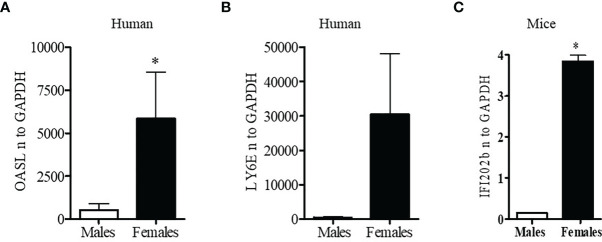
Healthy females have significantly higher interferon-related genes. Peripheral blood mononuclear cells (PBMC) (1-2 x10^6^ cells) were isolated from healthy males (n=5) and females (n=5) and RNA was isolated. 100 ng of RNA was used for real-time PCR analysis of OASL **(A)** and Ly6E **(B)**. **(C)** Female BWF1 mice have increased IFI202b gene expression. Splenocytes were obtained from 8-10-week-old male and female BWF1 mice (n=2), RNA isolated, and one-step real-time PCR performed with 100 ng of RNA from each sample. All values were normalized to GAPDH levels. *p < 0.05.

Since lupus is a gender biased disease with female to male ratio of 9:1, we were interested to see whether expression of interferon-related genes was different in healthy male and female volunteers. Peripheral blood mononuclear cells (PBMC; 1-2 x10^6^ cells) were collected, lysed, RNA isolated, and real-time PCR performed with human *LY6E, OASL*, and *GAPDH* specific primers and probes (Applied Biosystems, Foster City, CA, USA). These genes were selected because their expression levels in SLE patients are strongly correlated with SLEDAI (SLE disease activity index) scores (39, 40). We found that healthy females have significantly higher levels (5-10-fold) of the interferon-regulated gene (*OASL*) compared to healthy males (**Figure 1A**). We also found that *LY6E*, another interferon-regulated gene, was increased in healthy females compared to healthy males (**Figure 1B**). These data suggest that healthy females have higher interferon-related gene signature compared to healthy males.

### Female BWF1 Lupus Mice Have Significantly Increased Expression of Interferon Genes Compared to Age and Sex Matched Males

To better understand the gender-based differences in expression of a type one-interferon-induced gene (*IFI202b*) between the sexes, we isolated splenocytes from age matched male and female lupus prone NZBxNZW (F1) (BWF1) mice. RNA was isolated and real-time PCR was performed with gene specific primers and probe for *IFI202b*. We found that female BWF1 mice had significantly increased expression of *IFI202b* compared to male mice (**Figure 1C**). These data suggest that gender-based differences of interferon-inducible gene (*IFI202b*) expression in lupus has clinical significance since *IFI202b* has been identified as a lupus susceptibility gene (41–43).

### SLE Patients Have Increased Levels of IL-6, IL-17, and IL-21 Pro-Inflammatory Cytokines. Plasma Estradiol Levels Positively Correlates With IL-6 and IL-21, and Plasma IL-21 Levels With SLE Disease Activity Index (SLEDAI) Scores.

**Figure 2:**
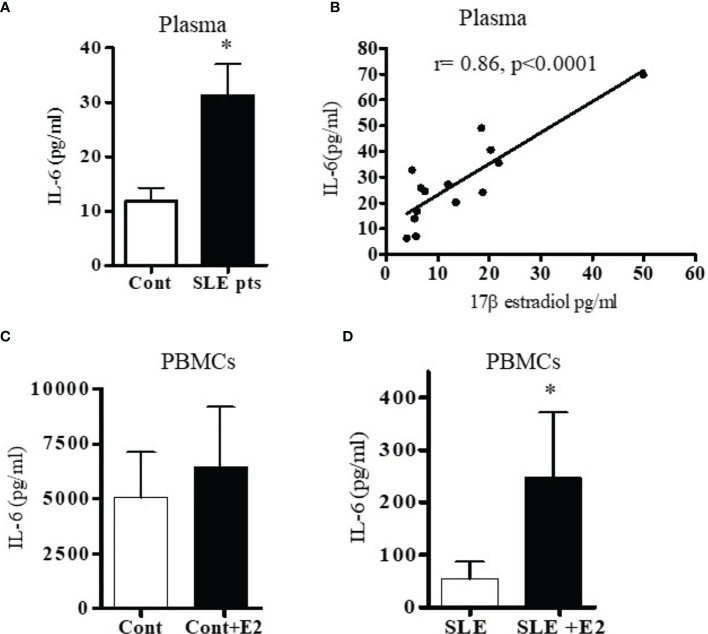
SLE patients have increased levels of the pro-inflammatory cytokine IL-6. Plasma estradiol positively correlates with IL-6. 17β-estradiol increased IL-6 levels in both healthy control and in SLE patients’ PBMCs. **(A)** Plasma levels of IL-6 were measured in female SLE patients (n=13) and in healthy controls (n=11) by ELISA. **(B)** Correlation between plasma 17β-estradiol levels and IL-6 in SLE patients (n=14), (p < 0.0001). PBMCs of healthy controls, n=11 **(C)** and SLE patients, n=11 **(D)** were cultured with 17β-estradiol (50 pg/ml) for 24-48 h and supernatants measured by multiplex assays. *p < 0.05.

**Figure 3:**
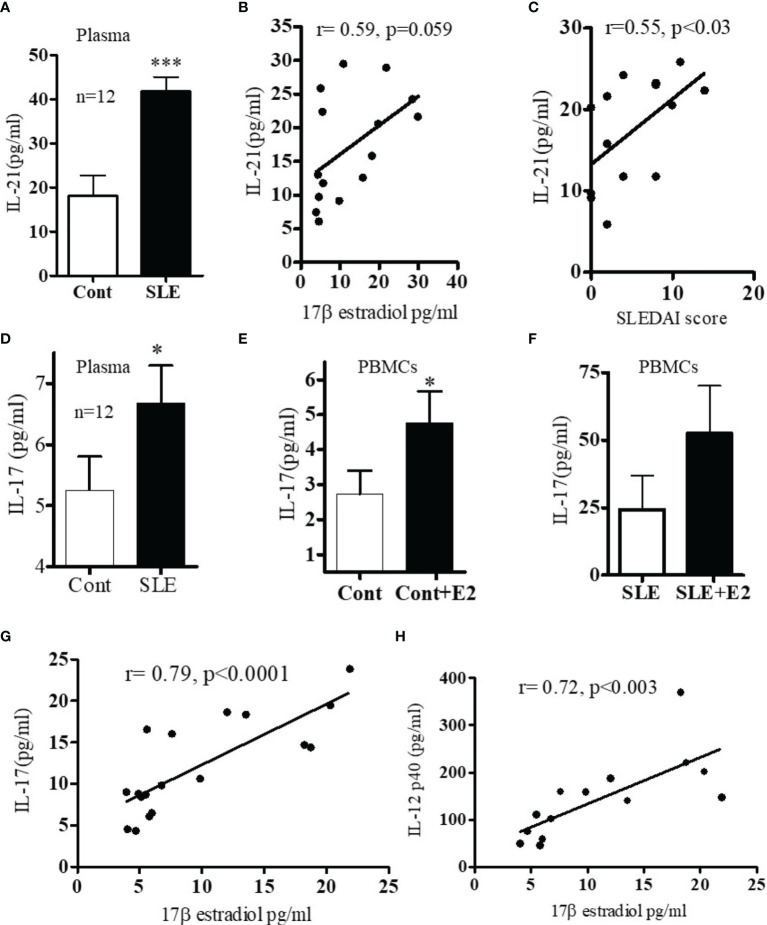
**(A)** SLE patients have increased levels of IL-17 and IL-21 pro-inflammatory cytokines. Plasma estradiol levels positively correlates with IL-21. **(A)** IL-21 levels in healthy controls (n=12) *vs.* SLE patients’ (n=12) plasma. **(B)** Correlation between plasma estradiol levels and IL-21 in SLE female patients (n=15). **(C)** Correlation between IL-21 levels and SLEDAI (SLE disease activity index) score in SLE female patients (n=14). **(D)** IL-17 protein levels in healthy controls (n=12) *vs* female SLE patients’ (n=12) plasma. **(E)** IL-17 protein levels measured from supernatant of PBMCs of healthy (n=11) controls PBMCs culture *vs* healthy controls PBMCs+E2 (50 pg/ml). **(F)** IL-17 protein levels of SLE (n=11) female patients PBMCs *vs* SLE patients PBMC+E2 were cultured with 17β-estradiol (50 pg/ml) for 24-48 h and supernatant was measured by multiplex assay (MAP) for IL-17. **(G)** Correlation between plasma estradiol levels and IL-17 in female SLE patients (n=18). **(H)** Correlation between plasma estradiol levels and IL-12-p40 in female SLE patients (n=14). *p < 0.05, ***p < 0.001.

Because both clinical and genetic polymorphism studies indicate roles of IL-6 (44), IL-17 (45, 46), IL-12/23 (47, 48) and IL-21 (49–52) in SLE pathogenesis, we chose these genes for analysis. We first analyzed IL-6 levels in plasma and found that IL-6 was significantly increased in SLE patients compared to healthy controls (**Figure 2A**). Interestingly, we also found significant positive correlations between plasma levels of estradiol and IL-6 in SLE patients (**Figure 2B**). Further, we found that *in vitro* cell culture of PBMCs from both SLE patients and healthy controls treated with 17β-estradiol at physiological concentration (50 pg/ml) increased secretion of many pro-inflammatory cytokines including IL-6 in the healthy control group (**Figure 2C**). The increase in the SLE group was also significant for IL-6 (**Figure 2D**). We also found that IL-21 levels in plasma of SLE patients was significantly increased compared to healthy controls (**Figure 3A**) and the IL-21 level positively correlated with plasma levels of estradiol (**Figure 3B**). Importantly, we also found positive correlation between plasma IL-21 and SLEDAI (SLE disease activity index) score in SLE patients (**Figure 3C**). Furthermore, we found that plasma IL-17 levels were significantly increased in SLE patients compared to healthy controls (**Figure 3D**). In addition, we noted that secreted IL-17 levels were significantly increased when healthy controls PBMCs were cultured with 17β-estradiol (50 pg/ml) (**Figure 3E**). However in SLE patients, the secreted level of IL-17 did not reach the significance threshold (**Figure 3F**). Further, we found that plasma level of 17β estradiol positively correlated with plasma levels of IL-17 and IL-12 p40 protein (**Figures 3G, H**).

These data indicate that 17β-estradiol influences many pro-inflammatory cytokines in both healthy controls and in SLE patients albeit differentially. This differential response may be due to environmental milieu in SLE patients’ immune cells. However, future detailed study will be required to pin-point the exact mechanisms.

### Plasma Levels of Various Pro-Inflammatory Cytokines/Chemokines Increased in SLE Patients Including Interferon (IFNγ), Interleukins (IL-18, IL-23), and Stem Cell Factor (SCF)

**Figure 4:**
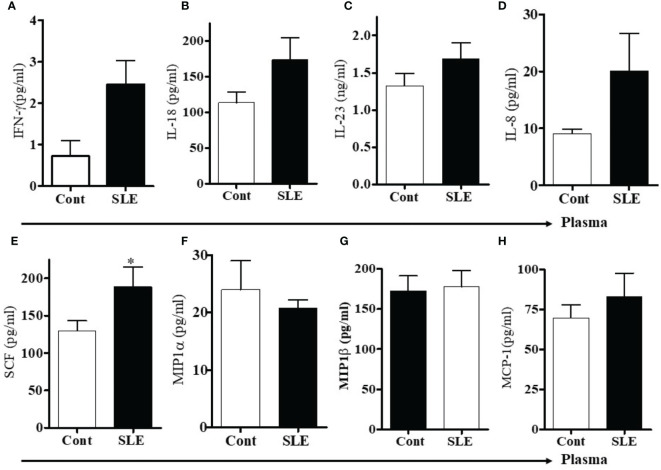
Plasma levels of pro-inflammatory cytokines, chemokines including stem cell factor (SCF) were increased in SLE patients. To address the differences of pro-inflammatory cytokines and chemokines between healthy controls and SLE patients, plasma levels of pro-inflammatory cytokines, chemokines and stem cell factor (SCF) were measured in healthy controls (n=10-12) and SLE patients (n= 10-12) by RBM multiplex assay. **(A)** IFN-γ, **(B)** IL-18, **(C)** IL-23, **(D)** IL-8, **(E)** Stem cell factor (SCF), **(F)** MIP1α, **(G)** MIP1β, and **(H)** MCP-1. *p < 0.05.

To measure levels of pro-inflammatory cytokines and chemokines, we used multiplex RBM to analyze the plasma of SLE patients and healthy controls. We found that protein levels of IFNγ, IL-23, and IL-18 were increased in SLE patients compared to healthy controls (**Figures 4A–C**). In addition, we found that the level of IL-8 was increased and SCF was significantly increased in SLE patients (**Figures 4D, E**). The levels of MIP1-α and MIP1-β were comparable between healthy controls and SLE patients (**Figures 4F, G**). However, the level of monocyte chemotactic protein-1(MCP-1) was slightly increased in SLE patients (**Figure 4H**). These data suggest that lupus patients do indeed have increased plasma levels of many pro-inflammatory biomarkers.

### 17β-Estradiol Increases Pro-Inflammatory Cytokines and Chemokines in Both Healthy Control and SLE Patients’ PBMCs

**Figure 5:**
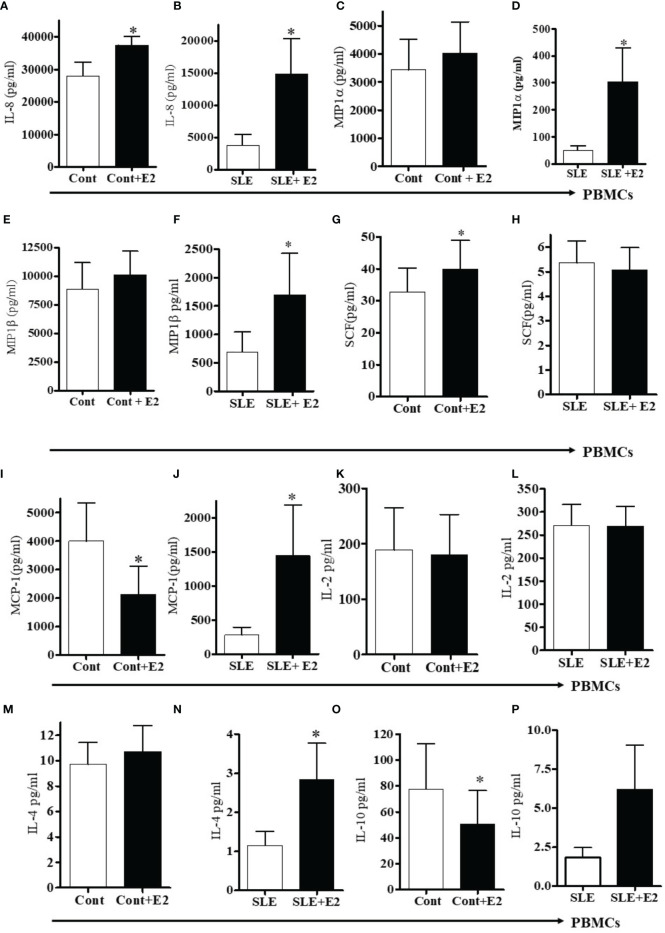
17β-estradiol increases pro-inflammatory chemokines in both healthy control and SLE patients’ PBMCs. PBMCs of female healthy controls and female SLE patients were cultured with 17β-estradiol (50 pg/ml) for 24-48h range (n=10-12). Culture supernatants were obtained and levels of IL-8 **(A, B)**, MIP1α **(C, D)**, MIP1β **(E, F)**, SCF **(G, H)** MCP-1 **(I, J)**, IL-2 **(K, L)**, IL-4 **(M, N)**, and IL-10 **(O, P)** were measured by multiplex assays (MAP). Conditions: **(A, C, E, G, I, K, M, O)**. Healthy female control cells (PBMCs), Healthy female control cells + E2 (50 pg/ml) n=11, **(B, D, F, H, J, L N, P)**. SLE female patient cells (PBMCs), SLE female patient cells + E2 (50 pg/ml) n=10. *p < 0.05.

17β-estradiol treatment differentially increased several pro-inflammatory chemokines and cytokines *in vitro*. We found increased level of IL-8 in plasma of SLE patients compared to healthy controls (**Figure 4D**). In addition, culture supernatant of PBMCs treated with 17β-estradiol in both healthy controls and SLE patients showed significantly increased secretion of IL-8 protein (**Figures 5A, B**). Plasma levels of MIP1α and MIP1β were comparable in control *vs* SLE patients (**Figures 4F, G**); however, both MIP1α and MIP1β levels were significantly increased after 17β-estradiol treatment (**Figures 5D, F**) in SLE patients’ but not in healthy control (**Figures 5C, E**) PBMCs *in vitro.* We found that fold increases were much higher in SLE patients’ cells compared to healthy control cells. We also found that 17β-estradiol significantly increased the SCF (stem cell factor) levels in healthy controls cells (**Figure 5G**); however, in SLE patients, there were no level changes detected (**Figure 5H**). Interestingly, we found that MCP-1 levels were significantly decreased with the treatment of 17β-estradiol in healthy control cells (**Figure 5I**), in contrast to SLE patients’ cells in which 17β-estradiol treatment significantly increased MCP-1 levels (**Figure 5J**). Thus, 17β-estradiol-induced changes in MCP-1 level was bidirectional. We also determined levels of other cytokines such as (IL-2, IL-4, and IL-10). Our data showed no change in IL-2 levels in both control and SLE PBMCs treated with 17β-estradiol (**Figures 5K, L**). We also found that no change in control PBMCs levels for IL-4; however, an significant increased secretion of IL-4 in SLE patients’ PBMCs (**Figures 5M, N**). Of significance, we also found that IL-10 levels were significantly decreased in control PBMCs (**Figure 5O**) and increased in SLE patients PBMCs treated with 17β-estradiol (**Figure 5P**). These changes were dynamic and further indicates differential effects of 17β-estradiol on various cytokines in healthy control *vs* SLE patients’ PBMCs. In addition, we noted that the absolute value of levels of various chemokines were higher in the healthy control compared to SLE patients groups before 17β-estradiol treatment (**Figure 5**). This may be due to differences in the diseased state or environmental milieu in healthy *versus* diseased cells (SLE), where defects in the SLE patients’ immune cells including differences in anergy and unresponsive state, prevent these cells from responding as well as healthy control cells in the secretion of various cytokines-chemokines. Additionally, a recent report suggests that SLE patients’ cells either enter an exhausted state or become tolerant to stimulation for cytokine production as the disease worsens (53). Altogether, these data indicate that 17β-estradiol has differential effect in healthy control and SLE patients’ cells and participates in the induction of pro-inflammatory cytokines and chemokines in both control and SLE patients, but at a much higher level in SLE patients.

### Estradiol Increases Pro-Inflammatory Cytokine Levels of IFN-γ, IL-18, and IL-23 in SLE Patients’ PBMCs Compared to Healthy Controls

**Figure 6:**
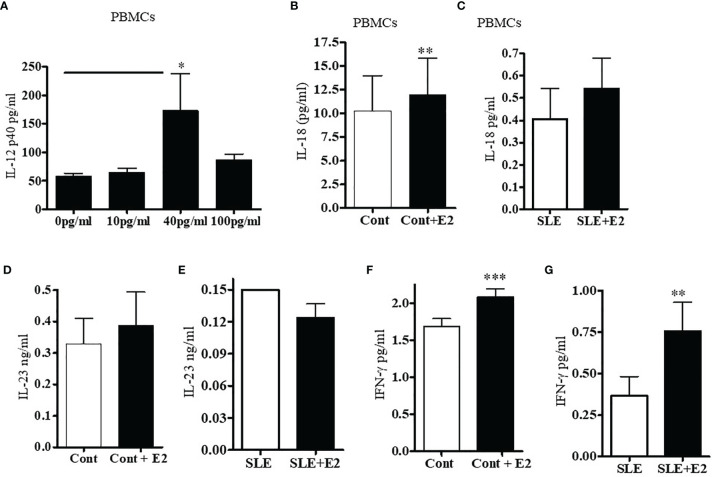
17β-estradiol increases pro-inflammatory cytokines IL-12p40, IL-18, and IL-23 in SLE patients compared to healthy controls. PBMCs from female SLE patients (n=7) were obtained and 2-4x10^6^ cells were cultured with 17β-estradiol (E2 at 10, 40 and 100 pg/ml) for 24-48 h. Culture supernatants were obtained. **(A)** IL-12p40 levels were measured with ELISA (BioLegend). *p < 0.05. **(B)** IL-18 levels were measured in the supernatant of healthy control PBMCs treated with E2 (50 pg/ml) (n=11). **(C)** IL-18 levels were measured in the supernatant of SLE patients PBMCs treated with E2 (50 pg/ml) (n=11). **(D)** IL-23 levels were measured in the supernatant of healthy control PBMCs treated with E2 (50 pg/ml) (n=8). **(E)** IL-23 levels were measured in the supernatant of SLE patients PBMCs treated with E2 (50 pg/ml) (n=9). **(F)** PBMCs (4x10^6^) of healthy controls (n=9) were cultured with 17β-estradiol (50 pg/ml). Culture supernatants were obtained after (24-48 h) and the level of secreted IFNγ was measured with multiplex assay (RBM-MAP, Austin, TX, USA). *p < 0.05. **(G)** IFNγ levels were measured in the supernatant of SLE patients PBMCs treated with E2 (50 pg/ml) (n=8). *p < 0.05, **p < 0.01, ***p < 0.001.

We were interested to see whether estradiol regulates pro-inflammatory cytokines in SLE patients’ PBMCs. Lupus patients’ PBMCs were isolated and cultured with a range of 17β-estradiol for 24-48 h (10, 40 and 100 pg/ml) to cover the physiologic plasma levels characteristic of menopause through the highest levels in the menstrual cycles. Culture supernatants were obtained and the level of IL-12p40 was measured by ELISA. We found that 17β-estradiol at mid-cycle levels (40 pg/ml) significantly increased levels of IL-12 in the supernatant (**Figure 6A**). However, at 100 pg/ml, we did not find further increase of IL-12p40; thereby indicating that the optimum dose for maximum increase of IL-12p40 is ~40-50 pg/ml. Further, we found that 17β-estradiol treatment (50 pg/ml) of healthy PBMCs significantly increased secreted IL-18 protein level in the supernatant (**Figure 6B**). We also found increased IL-23 secretion in healthy control PBMCs after 17β-estradiol treatment (**Figure 6D**) but it did not reach the significance level. There were no significant changes in levels of IL-23 secreted from SLE patients’ PBMCs after 17β-estradiol treatment (**Figure 6E**). Thus, our data showed differential effect of 17β-estradiol on IL-18 and IL-23 expression/secretion. Importantly, we found that 17β-estradiol treatment of healthy control and SLE patients (PBMCs) significantly increased the production of IFNγ (**Figures 6F, G**). Taken together, these data indicate that estrogen participates in the induction of pro-inflammatory cytokines and interferon genes.

### 17β-Estradiol Increases Levels of CD3^+^CD69^+^ and CD3^+^IFNγ^+^ T Cells in SLE Patients’ PBMCs

**Figure 7:**
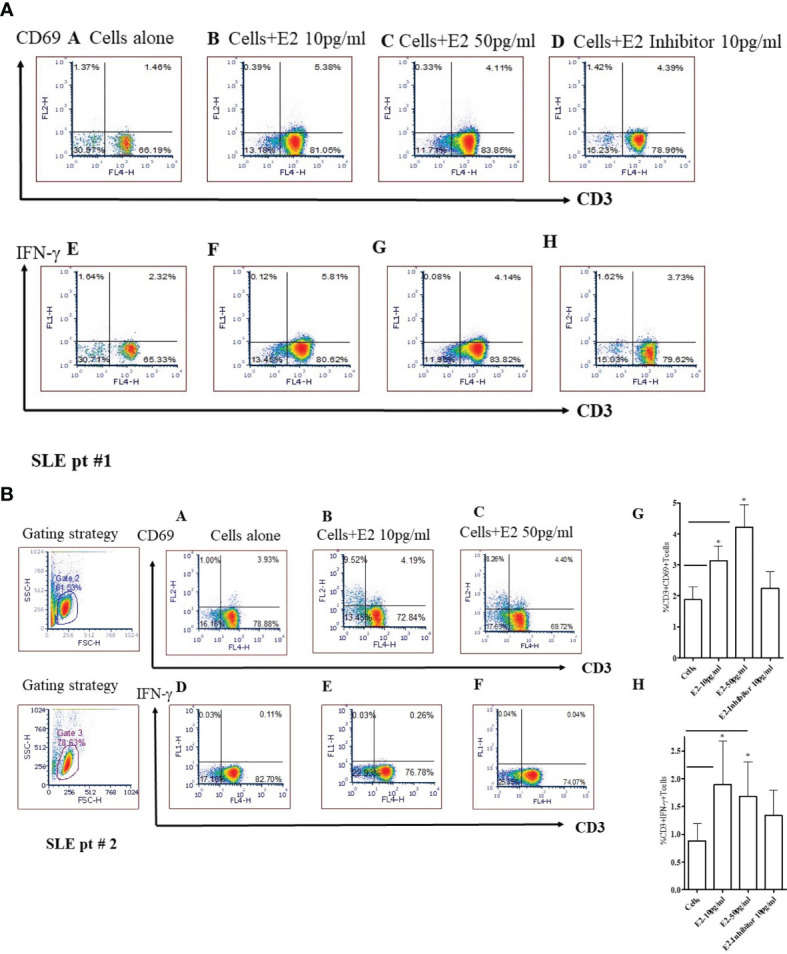
17β-estradiol (E2) increases the percent expression of CD3^+^CD69^+^ and CD3^+^IFNγ^+^ T cells in SLE patients. Female SLE patient peripheral blood mononuclear cells (2x10^6^ cells) were obtained and cultured for 24 hours in serum-free media. Cells were stained with anti-CD3, anti-IFNγ and, anti-CD69 Abs and analyzed by FACS. For intracellular IFNγ staining, cells were first fixed, permeabilized (as per manufacturer’s protocol; BD Biosciences, San Jose, CA) and stained. Cells were washed two times with PBS and data acquired at the UCLA Flow Cytometry Core Facility. Data were analyzed using FCS Express™ De Novo software (Thornhill, Ontario, Canada). SLE pt #1. **Figure 7A**: CD69: Panels **(A)**: Cells alone; **(B)**: Cells + E2 (10 pg/ml); **(C)** Cells + E2 (50 pg/ml); **(D)** Cells + E2 (10 pg/ml) + E2 inhibitor - estrogen receptor alpha antagonist, ICI-182780 (10 pg/ml). IFN-γ: **(E)** Cells alone; **(F)** Cells + E2 (10 pg/ml); **(G)** Cells + E2 (50 pg/ml); **(H)** Cells + E2 (10 pg/ml) + E2 inhibitor (10 pg/ml). SLE pt #2. **Figure 7B**: CD69: Panels **(A)** Cells alone; **(B)** Cells + E2 (10 pg/ml); **(C)** Cells + E2 (50 pg/ml). IFN-γ: Panels **(D)** Cells alone; **(E)** Cells + E2 (10 pg/ml); **(F)** Cells + E2 (50 pg/ml), **(G)** Combined data for CD3^+^CD69^+^ from SLE patients (n=8). *p < 0.05. **(H)** Combined data from SLE patients (n=6) for CD3^+^IFNγ^+^ T cells; *p < 0.05.

To test whether 17β-estradiol treatment alters T cell activation, we cultured PBMCs from SLE patients with a physiological concentration of 17β-estradiol in serum-free media for 24 hours. FCS (fetal calf serum) is known to have varied levels of endogenous androgen and other factors that may influence E2-PBMC culture data (54). Therefore, we used serum-free media to avoid further complexity in the results. As shown in **Figure 7**, we found that 17β-estradiol at 10-50 pg/ml concentrations significantly increased the percentage of CD3^+^CD69^+^ [**Figures 7A, B** (Panels B, C) and CD3^+^IFNγ^+^ **Figures 7A, B** (Panels F, G)] T cells; whereas, addition of an inhibitor of the ER-α prevents this effect [**Figure 7A** (Panels 7D, H) and **Figure 7B** (Panels G, H)]. Thus, our data indicates that 17β-estradiol increases CD69 expression and IFNγ production in T cells of SLE patients.

## Discussion

The present study was designed to identify and validate estradiol-regulated candidate genes that may be responsible for lupus development in females. The specific interferon genes were chosen based on their high expression in most patients with SLE (39, 40). We provide evidence that 17β-estradiol regulates IFN genes differentially in healthy controls and in SLE patients. We demonstrated significant increased protein level of secreted IFN-γ after 17β-estradiol treatment. Further, we showed that SLE patients have increased plasma concentrations of IL-6, IL-17, and IL-21 pro-inflammatory cytokines compared to healthy controls. Our data for IL-6 was in agreement with other investigators who have found similar increased IL-6 level in active SLE patients (55–57). Herein, we showed that 17β-estradiol further increased protein level of IL-6 in healthy control cells (**Figure 2**) and thus plays an important role in SLE disease pathology. Lupus nephritis patients have increased level of urinary IL-6 and expression of IL-6 was increased in the glomerular tissues (58–60). Further, plasma estradiol levels were positively correlated with levels of IL-6, IL-12 p40, IL-17, and IL-21 in our data. To our clinical significance, we found that plasma level of IL-21 also positively correlated with SLEDAI score of SLE patients.

We demonstrated in this study that 17β-estradiol increases CD3^+^CD69^+^ and CD3^+^IFNγ^+^ T cells in SLE patients and that 17β-estradiol treatment increases secreted IFNγ protein levels in healthy control PBMCs. Thus, 17β-estradiol plays a significant role in diverting immune responses toward pro-inflammatory pathways. Our data suggests that increased levels of 17β-estradiol may contribute to the female predisposition to SLE partly through the effects of the hormone on pro-inflammatory pathway activation.

The molecular interaction between pro-inflammatory pathways and 17β-estradiol in SLE remains to be fully elucidated. Moreover, the molecular mechanisms by which 17β-estradiol interacts with IFN and T cells, B cells, and antigen presenting cells (APC) in SLE is poorly understood. In the present study, we showed that 17β-estradiol treatment increased the expression of early activation marker CD69 and IFNγ on CD3^+^ T cells (**Figure 7**). Others have shown increased numbers of CD4^+^CD69^+^ T cells with altered expression of interleukins and suggested a correlation with loss of self-tolerance in lupus mice (61). Of clinical significance, a human study in lupus patients also found that CD69^+^ T cells are increased and defective in function (62). Further, we demonstrated that an antagonist of the estrogen receptor alpha (ER-α inhibitor) significantly decreased the expression of CD69 and IFN-γ in those T cells. In agreement with our study, Walters et al. (63) showed that estradiol targets the T cell signaling pathways in SLE and that the interferon-α pathway is upregulated in response to estradiol in SLE T cells (63). Previous reports have shown that ER-α was required for toll-like receptor (TLR)-induced stimulation of IL-23R expression, which may have effects on T cells and dendritic cells involved in the IL-23/IL-17 inflammatory pathway (64). Although, we did not evaluate the effect of 17β-estradiol on B cells and antigen presenting cells (such as dendritic cells and monocytes), previous studies have addressed these cell types (13, 65, 66) and showed enhancing B cell activity with increased IL-10 expression in monocytes and increased IFNγ production in dendritic and NK cells. In our studies, we tested the effect of 17β-estradiol treatment on PBMCs from healthy individuals and found that 17β-estradiol significantly increased production of IL-17 (**Figure 3**). In agreement with our study, previous studies have shown that 17β-estradiol induces IL-17 in lupus mice (67, 68). Thus, our data suggest that 17β-estradiol contribute to enhanced pro-inflammatory (Th17-IL-17) activation and stimulates the interferon pathway.

Recently, a connection between IL-23 and IL-17 has been identified (69). It was postulated that IL-23 promotes signal transducer and transcriptional activator 3 (STAT3) phosphorylation by Janus kinase 2 (JAK2) and tyrosine kinase 2 (TYK2) by binding to its receptor IL-23R (70, 71). IL-23 has also been shown to enhance the expression of retinoic acid receptor-associated orphan receptor γt (RORγt), which is involved in the expression of IL-17 and other Th17 cytokines (69, 72). We also showed herein that when SLE patients’ PBMCs were cultured with 17β-estradiol, IL-12 protein level increased (**Figure 6**). The role of IL-12 and the IL-23/Th17 axis has been recently demonstrated in lupus (73). Higher levels of the IL-12p40 subunit and circulating frequencies of Th17 cells were found to be correlated with SLE disease activity index (SLEDAI) including lupus nephritis (74, 75). A recent study described that the molecular interaction between IL-12 and IL-12R stimulates JAK2 and TYK2 activity, leading to the phosphorylation of STAT family members including STAT1 and STAT4 (73, 76). In addition, genetic polymorphisms within the IL-12/IL12R pathways have been associated with SLE pathogenesis (77, 78). Additionally, IL-17 has been shown to significantly induce B cell proliferation and antibody production synergistically with B cell activating factor (BAFF) (79–82). Thus, the cells which were exposed to 17β-estradiol may potentiate the inflammatory pathways through the IL-12/IL-23 and IL-17-Th17 axis.

The biologic effects of 17β-estradiol are mediated by binding to its receptors, ER-α and ER-β, expressed in many tissues including most immune cells (83–86), and by the membrane-bound G protein-coupled receptor, GPR30 (87). The role of 17β-estradiol on CD4^+^ and CD8^+^ T cells has been described recently (88–90). ER-α-specific signaling has been shown to be pro-inflammatory in T cells (91), leading to increased expression of the Th1-associated transcription factor Tbx21 (Tbet) (92) and enhanced production of IFNγ (93, 94). We found increased expression of CD69 and IFNγ in the CD4^+^ T cells when healthy controls or SLE patients’ PBMCs were cultured with 17β-estradiol (data not shown and **Figure 7**). More recently it was demonstrated that 17β-estradiol upregulates the cyclic AMP response element modulator alpha (CREM-α) protein, which down-regulates the production of IL-2 in human T cells (95). Differential expression of 17β-estradiol receptors in women with SLE has also been reported (96). In men with SLE, imbalances in estrogens and androgens can contribute to increased susceptibility to the active disease (97–101). Furthermore, administration of 5-dehydroepiandrosterone (DHEA) has been demonstrated to reduce disease activity in women with SLE (102).

We showed recently that plasma 17β-estradiol levels are significantly increased in female SLE patients compared to healthy females (30). Furthermore, we found that estradiol increases pro-inflammatory cytokines and chemokines (IL-8, IL-18, IL-23, CXCL1-7, MIP1α, and MIP1β), and level of estradiol positively-correlated with expression of pro-inflammatory cytokines and chemokines in SLE patients and with the levels of serum/plasma IL-6, IL-18, and IL-21/23 in SLE patients (**Figures 2B**, **3B**, **4**, **5**, and **6**). Our finding of increased level of IL-18 and the role of 17β-estradiol in further enhancing level of IL-18 in SLE patients has translational significance as recent studies have shown the impact of functional polymorphisms in SLE disease pathogenesis (103, 104). Together, these data indicate that 17β-estradiol increases the expression of interferon genes and pathways and thus could promote susceptibility to the disease in women. Thus, our data suggest that women may be more susceptible than men to SLE and other autoimmune diseases in part because many healthy women have higher base-line levels of interferon-regulated genes. However, future studies to delineate the detailed molecular mechanisms are required to address which signal transduction pathways are involved.

## Data Availability Statement

The raw data supporting the conclusions of this article will be made available by the authors, without undue reservation.

## Ethics Statement 

The study was reviewed and approved by the University of California, Los Angeles, Institutional Review Board (UCLA-IRB). The patients/participants provided their written informed consent to participate in this study. The animal study was reviewed and approved by the Animal Research Committee (ARC), University of California, Los Angeles.

## Author Contributions

RS contributed to the experimental design, obtaining funding, conducting experiments, analyzing data, preparing figures, and writing of the manuscript. BH contributed to funding and editing of the manuscript. DB contributed to figure and manuscript editing. All authors contributed to the article and approved the submitted version.

## Funding

This work was supported by the NIH grants AR54034, AI 083894, AI65645 to RS; UCLA Senate Core Grant to BH and RS; UCLA Oppenheimer Clinical Seed Grant and American Autoimmune Related Disease Association grant to RS.

## Conflict of Interest

The authors declare that the research was conducted in the absence of any commercial or financial relationships that could be construed as a potential conflict of interest.

## Publisher’s Note

All claims expressed in this article are solely those of the authors and do not necessarily represent those of their affiliated organizations, or those of the publisher, the editors and the reviewers. Any product that may be evaluated in this article, or claim that may be made by its manufacturer, is not guaranteed or endorsed by the publisher.
